# Facilitating non-tokenistic user involvement in research

**DOI:** 10.1186/s40900-019-0153-3

**Published:** 2019-06-04

**Authors:** Grace Inga Romsland, Kate Louise Milosavljevic, Tone Alm Andreassen

**Affiliations:** 1 Sunnaas Rehabilitation Hospital, Bjørnemyrveien, NO-1450 Nesoddtangen, Norway; 2 Faculty of Health Sciences, OsloMet - Oslo Metropolitan University, PO Box 4 St. Olavs plass, NO-0130 Oslo, Norway; 3 Centre for the study of professions (SPS), OsloMet - Oslo Metropolitan University, PO Box 4 St. Olavs plass, NO-0130 Oslo, Norway

**Keywords:** Patient participation, Rehabilitation, Research, Tokenism, User involvement

## Abstract

**Background:**

With the increase in user activism in the Western societies in recent years, there has also been an increase in promoting user involvement in research. Hence, is necessary to address the danger of tokenism, a false appearance of inclusiveness, in user involvement, as well as to explore methods for promoting active user involvement. Using a Norwegian research project on the rehabilitation processes following traumatic injuries organised via user involvement, this study reviews ways in which to avoid tokenism in user involvement and how to instead stimulate active user engagement in research.

**Methods:**

The analysis employs an ethnographic approach using participant observations from real life settings involving user involvement during the five years research process. The empirical material includes 472 pages of transcribed audio recordings from meetings between researchers and collaborators discussing personal experiences with traumatic injuries, and 340 pages of documents on the project’s involvement process. This empirical material was examined by thematic analysis, involving processes such as decontextualising, flagging and re-contextualising.

**Results:**

Two main categories of facilitation emerged as promoting non-tokenistic, active user involvement in research: 1) defining the collaborative arena, (i.e. the setting of collaboration) which entails preparing for participation and promoting active involvement, and 2) designing for research counselling, which involves gathering user perspectives and valuing criticism. Taking into account the existing asymmetric relationships between researchers and collaborators, enabling more evenly distributed power dynamics also proved to be essential.

**Conclusions:**

To achieve active participation that is relevant to the collaborators, two interconnected yet analytically independent themes should be considered: the collaborative arena and counselling. Both prove crucial for curbing power imbalance and stimulating the involvement process. The study indicates that non-tokenistic involvement should be anchored in the respect for participants and their ability to make contributions. This analysis can help researchers who seek active engagement and non-tokenistic involvement in research to find methods for facilitating and organising participation in their fields.

## Plain English summary

User involvement in research has increased in recent years. Therefore, the question of how to ensure valuable engagement and avoid tokenistic involvement organized just to look like real involvement needs to be addressed. Methods of promoting users’ active involvement must also be discussed. In this article we review ways of stimulating active user engagement in research. Our examination is based on a Norwegian research project about rehabilitation processes after traumatic injuries, which was organized with user involvement.

In the project, a group of service users collaborated with the researchers throughout the research process and contribute to the research with advice and experiences from living with traumatic injuries. We have used audio recordings of the meetings, meeting minutes and other documents from the collaboration to assess how active participation was promoted. Three researchers did the analyses. The collaborators commented and made input along the way.

The analysis showed that two types of arrangements had to be given attention: Firstly, the collaboration had to be prepared. Tools for active participation had to be planned and implemented. Secondly, opportunities for the collaborators to contribute to the research had to be created. This involved gathering user perspectives and valuing criticism. Common entities, such as jointly produced maps over research gaps, promoted the participation process. Addressing power differences also shown to be important for curbing power imbalance and stimulating the involvement process. Attitudes of respect from researchers toward collaborators who engage in user involvement seem to be crucial to the collaborators’ opportunity to contribute.

## Background

The desire for service user activism as well as self-determination and co-determination has increased in several parts of Western societies. Major funding bodies, such as the Research Council of Norway (RCN), have increasingly required user involvement to make up an integral part of research projects.

Previous analyses regarding processes of user involvement in health research have examined how to identify patient representatives who can be involved and engage them in the research process, which includes the benefits of involvement, as well as the disadvantages and barriers linked to patient engagement [1–7]. However, there is widespread concern that patient and public involvement remain tokenistic in nature, instead of truly valuing patient input and involvement [1, 8–11].

This study analyses user involvement in a Norwegian rehabilitation research project that intended to be non-tokenistic. The user involvement includes six health service users who contributed on the project, three of whom are men and three of whom are women. All collaborators reported experiencing severe traumatic injuries or being the next-of-kin for a seriously injured family member. Each collaborator had experienced a life-threatening accident, continued to experience severe impairments, and had undergone long-term rehabilitation.

The study explores methods for achieving non-tokenistic user involvement in research. ‘Tokenism’ is a term used to presenting the appearance of user involvement in decision-making when users do not actually have much influence. Tokenism can be understood as a false appearance of inclusiveness, indicating a gap between policy aims and actual practice [1, 11]. Tokenistic involvement somewhat resembles co-determination but does not include the participants holding any real influence [11, 12]. Tokenism can also occur if the abilities of the involved stakeholders are underestimated and the methods used to involve them are condescending [8, 13]. It can also occur if users find it difficult to add their experiential knowledge to the conversation with their contributions considered illegitimate in a discourse that often focuses on specialised scientific knowledge [11]. Therefore, tokenism can occur when participants of different backgrounds collaborate and they do not understand each other. Morrison and Dearden [11] use the term ‘representative artefacts’ to describe certain remedies used to overcome this challenge. This term describes objects or concepts that could represent the subject of the collaboration and can stimulate shared experiences, acted as a common tool for the participants regardless of different backgrounds. The use of specific objects (e.g. the meeting minutes for this study), can create opportunities for collaboration and bring participants together. Additionally, it is important to also note the concept of ‘language games’, the implicit rules for how, when, and what people speak. For example, if the researchers based their discussion on their common research interests, the other participants may find it difficult to express their ideas and opinions. The representational artefacts constitute one mechanism for establishing language games that can help involve individuals [11].

Non-tokenism requires a collaborative arena, which in this article refers to an area that is conductive to creating positive relationships, participation, and the possibility for collaborators to express their opinions and critiques.

There has been an increase in critical studies of user involvement practices in current research. McLaughlin [10] argues that too often, successful service-user involvement has been identified solely in terms of whether service users have contributed to the completion of a research project, while attention should also be paid to identifying when the process has been empowering and participatory or not. Furthermore, McLaughlin points out that it is unfair to expect service user co-researchers to contribute equally to all aspects of the research process. Forbat and Hubbard [14] show that service users risk low-quality performance when they are involved as co-researchers conducting interviews with other service users. The co-researchers in Forbat and Hubbard’s study constructed their identities as peers, but, despite training on how to manage interviews, they encountered difficulties from claiming certain experiences or opinions and rejecting their interviewees’ statements. Snow et al. [8] emphasise education and training but argue that universal ‘one-size-fits-all’ approaches may not enable diverse patient groups to participate. Instead, Snow et al. have developed a model that helps identify systematic barriers and power dynamics embedded in patient engagement processes. The model relates to the social location of the particular population of interest. Its core elements include a readiness assessment, strategies for identifying a suitable engagement method, planning engagement events, conducting gender-sensitive engagement, and evaluating the effect of engagement on planning.

In addition to such critical analyses, Morrison and Dearden [11] argue that relationships must be established via involving and empowering participants in such a way that they can contribute their knowledge. To achieve what Morrison and Dearden emphasise as ‘meaningful participation’, it is necessary to identify methods of involvement that allow users to express themselves in ways that are understandable, deemed valid by professionals, and contribute to the common process [15]. According to Maslin-Prothero, to promote the users contributions to research, researchers must ask questions about the most central areas, perceived from the users’ standpoint, and make sure both active and passive users are participating [16].

Morrison and Dearden point out that the development of a shared understanding is essential and can be achieved via participatory design and the creation of representational artefacts that can provide opportunities for participating and contribution [11]. Morrison and Dearden identify language as the key to mediating interaction and achieving meaningful participation. By promoting rules for the conversation, language games can prevent or promote the participation. Representational artefacts can be used to establish a language game that supports collaborative engagement and meaningful involvement.

Given that user involvement is crucial to high-quality research, tokenistic participation likely results in low-quality research. However, there is still limited research dedicated to identifying methods for achieving active engagement and non-tokenistic involvement. This study, therefore, aims to address this gap and identify measures that can facilitate non-tokenistic approaches to user involvement and stimulate active participation in research, which is relevant and valuable for all users involved. It should be noted that the empirical material is from a Norwegian rehabilitation research project.

## Methods

### Case of involvement: a user panel linked to a rehabilitation research project

The process of user involvement presently analysed stems from a research project that investigated transitions during rehabilitation following traumatic injuries. The research project investigated these transitions from specialised hospital-based rehabilitation to community-based rehabilitation and to employment services, seen from the perspectives of the professionals in these services, as well as from the perspectives of injured individuals in their process of biographical reconstruction [17]. User involvement in the rehabilitation research project was organised by establishing an advisory panel consisting of six collaborators who all reported personal experiences with spinal cord injuries, traumatic brain injuries, or multi-trauma, either as injured people, or what applied for two of them, as family members of injured individuals. All of them were active members of relevant Norwegian user organisations, and through this membership, displayed broad, general knowledge.

The aim of user involvement in the project was to ensure that the research was relevant to the challenges experienced by the collaborators. The goal of the analysis at hand was to gain comprehensive knowledge on non-tokenistic user participation in research and involved the researchers’ actions as well as how the collaborators participated. The project period lasted from 2013 to 2018, with the collaborators involved in the research process since its inception, via regular quarterly meetings. These meetings provided a setting for collaborating among users and researchers. Several topics were discussed, including the organisation of the panel’s involvement; the research methods, process, and problems; analyses and dissemination; and, finally, its content and conclusion.

This work involved the six collaborators, anonymised as C1 through C6, as well as the researchers, anonymised as R1 (project leader), R2 (project coordinator), R3 through R5 (three Ph.D. candidates, i.e. students researching to get the postgraduate degree Doctor of Philosophy (Ph.D.), and R6 through R11 (six senior researchers). All collaborators were generally present for each meeting; however, a minimum of a fifty-fifty distribution of collaborators and researchers, in favor of the collaborators, was required for each meeting to prevent the researchers from overpowering the collaborators. The project manager and project coordinator were always present, while the remaining researchers participated on a rotating basis. Meetings generally involved one or two Ph.D. candidates and two or three senior researchers.

Both the researchers, the Ph.D. candidates and the collaborators provided informed consent to have the meetings audio recorded and made available for analysis. Three of the researchers completed the work for this study, including analysis and writing the report. The collaborators participated in discussions about the analysis during the writing of the report, but did not join the three researchers in actually conducting the analysis. Indeed, for both researchers and collaborators, expectations are that it is challenging to be published in English-language journals as non-native English writers.

This study employs an ethnographic research approach which is a methodology for closely studying phenomena in the context in which they occur. Participant observation is often seen as a defining feature of this approach; furthermore, when the researchers themselves are also under investigation, the approach is referred to as auto-ethnography [18–20]. The ethnographic approach is deeply rooted in classical qualitative research methods, which are essentially exploratory. Health service research generally uses qualitative research methods to understand health and health-related issues from the patient’s point of view or within the context of human health, illness, suffering and healing [21]. This includes service user involvement and service user engagement with health research.

The data used for analysis stems from participant observations of cooperation between collaborators and researchers. The empirical material included audio recording of the meetings, meeting minutes, and other documents from the collaborative work. Each meeting lasted three hours, and included a meal. Meetings were held three or four times per year over a period of five years. Meeting minutes and memos, with one or two sentences reminding the leaders on practical tasks, summarised each meeting and provided direction for future collaboration and the research process.

Meeting minutes, meeting agendas and preparatory material (e.g. the researchers’ preparation for facilitating valuable user involvement), totalling 340 pages, were collected to compare planning with outcomes. While the meeting minutes served as a simple summary, the recorded discussions were rich in detail and depicted valuable involvement. These comparisons supported the analysis and showed e.g. needs for ‘interpretations’ of collaborators statements and anecdotes.

In all, 19 meetings were recorded and transcribed into 472 pages for analysis. The transcripts allowed for an analysis of the actual collaborative actions, rather than just the participants’ accounts of the collaboration, via interviews. These transcripts provided a rigorous analysis that was not only based on observation notes.

The three researchers reflexively worked regarding their own biases and attitudes. Challenges of being part of the research area were considered. An analytical distance was maintained through participation as a group, so the analytical process had a certain distance from each person’s lifeworld. Temporal distancing was also ensured, via the established time intervals between the meetings and the data transcription analysis. Nevertheless, the researchers’ presence in the group meetings was examined in order to provide insight into the overall atmosphere, and the understanding of the context of the data [21].

### The analytical approach

The analytical work was based on classical qualitative research analysis, employing thematic content analysis and processes for de-contextualising, flagging and re-contextualising concepts [22]. De-contextualisation involves finding meaningful ideas and examining beyond their original contexts. This tactic provides an opportunity for new interpretation and understanding. An example of such is sharing a meal, which was investigated to find new meanings beyond the collaborators being hungry.

Flagging refers to highlighting notions and concepts that can be used to search for significant theories that could be useful in developing new knowledge [21, 22].The researchers searched for concrete examples of research that could work positively in regard to the organisation of user involvement. The search was supported by Morrison and Dearden’s analysis, showing how representational artefacts could contribute to a common understanding of the work area [11]. The identified areas were assessed against the context and further investigated to discover complementary descriptions of relevant topics [22]. Words, concepts and meaningful content relevant to the purpose of the study were organised in such way that they could be coded, read, and reflected upon repeatedly [21]. This search for concepts and content helped condense and concentrate the data, making it easier to identify significant topics. Repeatedly condensing and comparing, which is essential in qualitative research [21], contributed to researcher insight, (e.g. the different meanings behind the common meal shared during meetings).

The researchers were all attentive to discovering aspects of phenomena of which they were not immediately aware. The search for ‘revelatory moments’ described by Trigger et al. [23] revealed unplanned, powerful and surprising episodes. One such example is the data showing how the collaborators could be critical of the researchers.

Finally, the re-contextualisation process meant that concepts and themes were probed to develop understandings of the various involvement techniques. Unpacking events, situations, and the contexts in which they occur [21] was crucial to developing an understanding of the factors that provide users with valuable participatory experiences in the research process. Condensing, coding, comparing and developing concepts all contributed to the design of patterns that showed how some concepts were important for collaboration while others were important for research counselling.

Although the requirements for generalisability in quantitative research do not apply to qualitative research, it is recognised that quality research conducted with transparency can provide new knowledge that may be applicable to similar cases. As pointed out by Moen and Middelthon [21], new concepts obtained from qualitative research can generally be comparable in certain contexts. Thus, it is argued that this applies to the present study.

## Results

This section presents the various measures used to facilitate non-tokenistic user involvement via two analytically distinct categories: ‘defining the collaboration arena’ and ‘designing for research counselling’. The first category encompasses facilitating the setting of the involvement, preparing for participation, and promoting engagement during the involvement process. The second category entails facilitating user involvement in certain aspects of the research process, deciding topics for discussion and valuing criticism. Within each of these categories, the analysis indicates the employment of different tools and approaches (Table 1).

**Table 1:** Categories of facilitation

Categories of facilitation	Defining the collaboration arena		Designing for research counselling	
Themes	Preparing for participation	Promoting active engagement	Gathering user perspectives	Valuing criticism
Approaches and measures	Accommodating collaborators' situations Ensuring a working relationship	Organising activities Disseminating research knowledge Handling language challenges	Deciding topics for reflection Translating experiences into implications for research	Stimulating independence Encouraging critical voices

While this analysis focuses on how researchers can facilitate user involvement, it is worth noting that the collaborators emphasised the importance of being effortfully engaged in the research process. One of the male collaborators, refered to as C1, said:

*When you as a user are involved on behalf of your organisation, you have a responsibility. The impact of user involvement depends also on the activity of the users. […] You cannot only place demands on the researchers.*

Norwegian non-government organisations have a tradition of cooperating with different authorities, and this tradition applies to Norwegian user organisations within the health services field. C1 expressed this cooperation by emphasising the requirements of collaborators. Thus, as the project evolved, the contribution of user involvement to the research was realised as a joint process.

### Defining the collaboration arena

#### Preparing for participation

‘Preparing for participation’ entailed both accommodating the situations of the involved collaborators and ensuring a working relationship between collaborators and researchers by implementing measures that made the collaborators feel recognised.

Specifically, accommodating the situations of the involved collaborators refers to arranging the collaborative arena’s adjustment to the needs of the involved collaborators. During the initial meeting, the user panel decided the day, time, and duration of the subsequent panel meetings. Because some collaborators had full-time jobs, the panel preferred to hold meetings in the evening. Several collaborators were also wheelchair users; therefore, the management team arranged for the meetings to be held in accessible facilities, with available parking nearby. To ensure a working relationship between collaborators and researchers, steps were taken to create a stimulating and somewhat informal collaborative environment wherein both groups could feel equal to each other.

Participants were welcomed to the meeting with a tasty evening meal, since several arrived straight from their daytime jobs. Sharing a common meal generated several meanings and became a common arena wherein researchers and collaborators alike could relax and chat informally. The meal was also a way for the researchers and collaborators to learn about each other and laid the foundation for a collaborative arena. The meal itself was an element that participants could commonly discuss which turned out as revelatory in the analysis. Each person added meaning to the meal and the meal linked the participants together.

Although unintended, the meal also worked as an artefact, linking the senior researchers, Ph.D. students, and collaborators across their different contexts. Representative artefacts may act as continuing features [11], and the meal became an important continuing feature that the entire group looked forward to. Overall, the shared meal produced pleasure and a sense of recognition that was essential to developing a collaborative arena.

The collaborators also received an honorarium per meeting. This honorarium was equal to the amount received for service user involvement in the Regional Health Authority, (i.e. Norwegian kroner equivalent to 40 € per hour). Transportation and accommodation costs were also covered. The collaborators emphasised the importance of payment as a symbol of recognising their equal participation with the researchers, and as a sign of the requirements for their active involvement. As C2, one of the female collaborators said, ‘*Active participation justifies economic compensation’*.

To accommodate cognitive impairments, P2, the project coordinator, assisted the panel by filling out the necessary paperwork. The agenda for the panel meetings, as well as the written preparatory material, was sent out two weeks in advance in order to accommodate cognitive impairments from injuries, as stated by C1:

*If things are new to me and require some kind of deep thinking and reviewing and so on, then I personally find it easier to come up with feedback if I, yes, I can sit and think it through for myself simply and wonder, and [...] some people express themselves better in writing than verbally.*

Additionally, because the collaborators were busy (with work, care and volunteer service), they needed to have the material available well in advance.

Furthermore, as the collaborators were invited via their user organisations, some preferred to have an opportunity to discuss the matters on the agenda with other members of their organisations. Guidelines for handling internal documents and materials had to be established to accommodate this desire.

The project coordinator compiled all written documents from the panel meetings in a ring-binder file for each participant. For those collaborators who preferred it, she also stored the binders and brought them already filled to the panel meetings in order to ease the participatory work of the collaborators struggling with cognitive impairments.

After every panel meeting, the project leader produced a written memo with the main points of the discussions, in addition to the ordinary minutes, as a way to create comprehensible and meaningful summaries. These memos were sent to the collaborators, the researchers, and the PhD students involved in the project.

The interpretation and ‘translation’ in the form of meeting minutes supported the common understanding of the current issues and could develop future opportunities for meaningful participation. This kind of perspective is an example of what can be accomplished through creating an appropriate representational artefact that is useful for denoting the topic of discussion [11]. Interpretations and summaries of the participant statements became significant artefacts that clarified input, transforming it into renewed knowledge.

As the collaborators were part-time participants, they asked for updates on the current stage of the research project, (e.g. ‘The data collection is concluded; currently, the data is being analysed’). These collaborators’ need for continuity resulted in a trial of the system wherein regular project updates were provided.

#### Promoting active involvement

Promoting active involvement entailed organising activities, disseminating research knowledge and handling language challenges. Taking into account the existing asymmetric relationships, enabling more evenly distributed power dynamics also was essential. This was achieved by organising active participation in the user panel.

Visual techniques to support an inclusive atmosphere and a positive tone were derived from participatory learning and action methods [15, 24, 25]. For example, during a workshop, collaborators and researchers were both instructed to identify their goals for the collaboration and what they might consider disappointing. Each person wrote his or her thoughts on sticky notes, which were collected anonymously and posted on two flip charts; one regarding goals and one regarding disappointments. Therefore, each participant could directly see the group’s overall expectations and concerns, and this served as the basis for a joint reflection. A large matrix, presented on a flip chart sheet, was also introduced, allowing the collaborators and researchers to develop separate themes regarding what they believed were important to the project. Each individual was given a certain number of sticky notes to divide among the topics, demonstrating their priorities. The notes were counted, and the results were presented to group.

With such approaches, linguistic skills are less essential, and the participants are more equal. Such methods used in research have the potential to promote mutual learning and development, as well as the cascade of learning and transfer of knowledge to others, leading to the co-creation of knowledge that is relevant, contextualised and useful to both the participant communities and producing research output [15]. Workshops employing various participatory approaches were used with the aim of evoking ideas and knowledge. In line with Kearney’s [15] view, this strategy led to the co-production of knowledge, with benefits for both researchers and service users. An example of this regards the areas that should be prioritised in user organisations in the future.

The meetings were often organised so all the participants could comment on one issue at a time, such as during a round-table discussion. This approach ensured that everyone’s voice was heard, since in a group setting, it is easy for some participants to dominate the conversation. Although a high level of engagement is desirable, this should not be at the expense of curbing others’ involvement. When participants have cognitive disabilities, facilitation may be particularly required to both those who need support to regulate their activity, and to encourage less dominant members to express themselves.

To reduce the risk of the collaboration being dominated by the researchers, who are most comfortable in the university setting, they took turns attending the meetings so that the user collaborators were never in the minority. Furthermore, the collaborators were encouraged to propose agenda items. The organising of the collaborators’ panel work was discussed as a result of their own initiative. By the request of some collaborators, meeting rooms and times were arranged in a way that offered the collaborators an opportunity to hold meetings on their own prior to the formal panel meetings.

Valuable and effective participation requires users’ access to research knowledge and information necessary for them to fulfil their mandate [8, 11, 16]. Observation transcripts displayed the exchange of information, opportunities to learn during the meetings, and researcher concerns about knowledge dissemination. Researchers R6 and R7 taught the topic of traumatic brain injuries. R8 and R9 presented an introduction to research methods, emphasising a simple description of qualitative methods. R1 provided information on the focus group method applied to the research project and the process of publishing papers in scientific journals.

Two types of language challenges appeared during the collaboration process: the use of English as an academic lingua franca and the use of academic terms in the analyses. A female collaborator addressed the problem in the following way:

*C3: For us ordinary people, […] it becomes so distracting and makes us feel a little lost when a lot of research language is used in the meetings here. When we try to ask and so on, and that is all very well, but I think… […] an important thing is to provide comprehensible explanations of the research, both to the outside world and to us, about the findings from papers that are presented here. To me, it is very important that it is translated into Norwegian. I spend a terribly long time reading in English.*

To minimise issues with English, written texts, documents and papers presented to the collaborators were translated into Norwegian, and used a popular science style as often as possible.

Several collaborators found the researchers’ academic terminology challenging. For example, when one of the young PhD candidates, R3, presented a draft from his research on inter-organisational collaboration, the discussion about the word ‘externalisation’ was a revelatory moment, illuminating the importance of the researchers’ language and conduct. The collaborators became frustrated, and the PhD student and the researchers had to explain the term, using more familiar terminology.

### Designing for research counselling

#### Gathering user perspectives

As emphasised in the application to the Research Council of Norway that provided funding for the project, the invitation to participate in the user panel stated that the panel would be involved in all the points of the research process to provide the best possible research results. As stated in the invitation, the project aimed to ensure the inclusion of topics important to those living with traumatic injuries. In addition, designing for research counselling involved taking the measures to implement this aim, gathering user perspectives, and valuing criticism.

Deciding topics for counselling involved researcher reflections on the topics presented by the collaborators’ meeting agendas (i.e., the aspects of the research process that could benefit from the collaborators’ perspectives). The researchers asked for advice on conducting the data collection and the overall study. Informational letters, interview guides, anonymised interview excerpts and preliminary analyses, were presented to the panel in order to collect collaborators’ thoughts, experiences, perspectives and recommendations.

The collaborators actively combined their acquired research knowledge with their experiences, highlighting themes and approaches that concerned them. They contributed to clarifying and specifying the tools for data collection. This included, for example, a vignette (a case description of an injured person) and the questions asked during focus group interviews with professionals in relation to the vignette:

*C4: [...] the questions from the case descriptions […] appear to be very relevant, but what I wondered a bit about was the questions on how they think he is doing personally. I think it would be important to ask, ‘How do you work? What are the challenges in a situation like this?’*

Thus, the collaborators influenced the Ph.D. student to become attentive to how the professionals handled this injured individual’s particular case.

Furthermore, the PhD candidates, who were inexperienced in meeting injured persons, sometimes required support during their interactions with the informants. During an interview conducted by R4, the informant started crying, and the collaborators advised R4 that she should not be scared but instead ask the informant whether he or she wanted to end the interview. A collaborator noted that tears were probably more frightening to the interviewer than the interviewee living with a traumatic injury.

The collaborators’ newly acquired research knowledge was put to practical use during the discussions on excerpts from the empirical material of the research project. For example, C2 attempted to perform analytical coding while reading the research data. This ‘professionalisation’ of the collaborators provided the necessary tool for discussing the research work, enabling the collaborators to participate in language games used in professional fields [11].

Interview guides and vignettes, other data materials and preliminary analyses served as representational artefacts that initiated new language games [11], allowing the participants to involve themselves in discussions. This led to reflections that could potentially result in outcomes different from those expected by the researchers. For example, in exploring the data from focus group interviews with professionals, collaborator closeness to service user conditions shifted the discussion toward the assessment of the professionals’ methods for handling service user problems. The responses of three collaborators are presented below:

*C2: Now we are in an open reading, right? [...] Because I just have to say I think they express so much uncertainty, both the physiotherapist and the occupational therapist and the case manager, in relation to the case. [...] There is such fumbling when it comes to return-to-work and that must be very important for him [the case person in the vignette], to be able to have such a goal, to return to work again, completely or part time.*

*C3: Yes, ‘fumbling’, I think that was a good description, […] but first of all, they demonstrate very little understanding. They do not possess the complete full interdisciplinary breadth and understanding, which I think is important in this case.*

*C4: Yes, yes. I actually agree. I think, how are all these professionals going to find one way – some fairly similar standpoint? [...] That’s what first comes to mind; how in the world are they going to be able to agree on good measures for the patient or the person in question? That’s – it’s almost a little scary, too, that there can be such different positions, given the same starting point.*

The collaborators appeared slightly upset on behalf of the patient in regard to the dialogue of the focus group interviews. Carric et al. point out that the different needs of users and researchers [2] will place different emphases on what should be important in the research approach. The fact that the collaborators were particularly concerned with the service users, even when discussing the interaction between the professionals, was an important factor that formed the basis of the overall analyses.

The meeting minutes emphasised for the collaborators that the presented excerpts comprised only a minor part of the interviews and that several topics were elaborated upon and require further analysis to examine whether researchers’ will be validated. The collaborators’ discussion highlighted the aspects that require attention in the analyses.

Collaborators’ personal experiences may lead to what deBrún et al. call ‘transformative moments’, wherein cooperation and dialogue may move a process further along [25]. One of the collaborators recalled the discussion on her encounters with the health service system:

*C5: ... I have been going for sixteen years with this (kind of relation to healthcare), when I relate this (discussion in interview excerpts) here, to the struggle I’ve had [...], I’m just so familiar with it (the discussion).*

Such statements helped the researchers understand the significance of what the collaborators regarded as important and contributed to the research process. Frequently, the collaborators’ contributions were related to the experiences of patients and service users; thus, the discussions could take the form of storytelling, which is a type of language game [11]. As a language game, the story regulates what is to be talked about. It can also function as a representative artefact. Participants and listeners can add different significance to the story, but the story remains a common narrative. Thus, the story can be capable of linking the narrators and demonstrating the importance of their experiences. Thus, representational artefacts can appear to be continuing features, driving the collaboration forward [11]. However, stories about the experiences of the injured do not in themselves contain recommendations relevant to the research project. Rather, they point to aspects deserving attention within the research process.

The implications for the research project were inferred from the opinions, statements and stories presented during the panel discussions. The project leader R1 managed interpreting and summarising the discussions so that the results could be conveyed in points that addressed important aspects of the emerging themes from the reflections.

Examining the themes voiced by the collaborators revealed a consistency in several recurring topics. Toward the end of the project period, R1 summarised these topics. Furthermore, to realise the value of user involvement in research, the ‘translation’ tasks of summarising, systematising, interpreting and inferring implications for research had to be undertaken.

### Valuing criticism

Valuing criticism refers to both stimulating independence on behalf of the collaborators and encouraging critical voices. The researchers facilitated independence via methods for promoting group dynamics and active participation [15]. For example, one technique, which sparked laughter and energy, but also had a deeper intention, was employed when collaborators and researchers were first introduced: All participants were instructed to introduce themselves by adding an alliterative adjective before their names (e.g. ‘I’m marvellous Mary’, ‘this is angry Amy’, or ‘I’m pretty Peter’, etc.). This ice-breaker exercise was used to create an informal atmosphere, with the collaborators highlighted as independent individuals with their own characteristics and opinions. Feeling visible in a safe and harmless setting can provide the basis for independence and the development of one’s own position. Another example occurred during a workshop, wherein the participants were asked to write their proposals on sticky notes, which were then placed on a whiteboard, arranged in thematic groups and discussed. This creativity-enhancing approach transformed anonymous posts into honest and open discussions with all the collaborators discussing different researcher results and drafts and thus demonstrating independence through critically evaluating debate inputs. Although the collaborators were experienced members and spokespersons for their Norwegian user organisations, they still benefited from these methods because they led to direct discussions.

The collaborators demonstrated the ability to articulate views that countered the researcher attitudes, choices, and analyses. The collaborators corrected the researchers, and R1 ensured that the users felt safe to express criticism and feedback when necessary. A revelatory example of this involves a senior researchers’ work in analysing components of the material. R10 presented an outline of an article that contained a critical analysis of processes in hospital settings, citing the fact that for some patients, the hospital becomes a safe haven, the professionals’ close relationships with the patients adding value to their work, and the setting can thus become too ‘cosy’. The collaborators and others laughed, but then collaborator C2 interrupted. The following dialogue ensued:

*C2: It is important to have a nice time; (many attendees laugh) I think that’s very important!*

*R10: Just the second point I raised ...*

*R1: ... if you want to comment on something, we can make time for that now.*

*C2: Yes, I just have to say that it is super important that you can have a nice time, that we meet friendly people who are decent and appear human, not just as a professional machine, you know. It has an effect in itself, meeting people who see you as a human being. I mean, it has a therapeutic effect of its own. Experiencing niceness creates energy; it creates a lot of good energy, I think.*

Criticising the researcher’s critical approach is possible here due to the collaborator’s unique experience with the need to be protected as a vulnerable patient in the hospital. Snow et al. point out that ‘activating’ patients and deploying them in involvement processes often places the onus on patients to learn how to interact with professionals in ways that conform to the professionals’ norms and reinforce the existing power dynamics [8]. In this research project, attempts were made to emphasise participants’ voices, as shown by the project leader’s rapid intervention to provide opportunities for comments. This attitude demands openness from researchers and contributes to empowerment but is also important for research. Listening to users’ voices, as demonstrated above, provides added depth to the critical analysis of hospital life. As R10 himself concluded:

*This is important. You have emphasised the significance of good relations between patients and therapists during long-lasting rehabilitation processes, that this is decisive to achieve results when one has to do one’s utmost.*

Overall, facilitating non-tokenistic involvement aimed to highlight all the needs of a user group in order to enable its members to valuable participating. Not all preparations for involvement were the results of researcher facilitation efforts alone. Rather, many of the steps were implemented as responses to requests from collaborators. Hence, to achieve participation significant for the involved, such as Morrison and Dearden’s [11] emphasise on ‘meaningful participation’, researchers should listen and respond to the needs expressed by users.

Signs of successful involvement in this study included the collaborators’ deep commitment to using the arrangements and their eager adaption of the measures for their own needs. The collaborators could both oppose and provide the researchers with the necessary countermeasures, but in the evaluations, they offered their support for the facilitation of user involvement. The fact that the collaborators lasted the entire project period, which spanned several years, could also have familiarised the collaborators with the research and the researchers, levelling the inequality as a result.

## Discussion

In keeping with McLaughlin’s findings [10], this analysis has shown that the nature of the involvement process is crucial for determining if participation has been successful and positive. This approach allows for the possibility to specify where the process has resulted in successful involvement and where it has failed to do so. We found the following measures successful in this study:

- Creating an informal and positive atmosphere via hospitality and sharing a meal.
- Introducing tasks that involve laughter and produce energy.
- Using methods that stimulate non-academic reflection and thinking, such as brain-storming, sticky notes attached to a wall or flip chart and matrices to present ideas, priorities or plans. These methods can also curb asymmetry.
- Educating participants about research topics and -methods.
- Being sensitive to collaborator problems, such as the need to discuss topics without researchers being present, language issues or equivalent inhibitory factors.
- Encouraging all participants to express themselves and ensuring that all voices are heard (e.g. by consecutively awarding time for speaking).
- Being attentive to the collaborator expressions, views and feedback and acknowledging the fact that collaborators are contributing because of their particular experiences and viewpoints.
- Facilitating space for participants to express opposition or critique concepts and ideas.
- Contributing to understanding and development by summarising and interpreting discussions and anecdotal statements.

Involving service users in health research means considering their situations. First, the reason for placing individuals in the positions of service users is that their lives are affected by illness, injury or impairment which can influence the involvement process. For example, cognitive impairment can affect concentration during meetings, and the choice of venue can influence access to the meeting room and facilities. Facilitating non-tokenistic user involvement means establishing a system of collaboration that allows service users to contribute in their desired capacity.

Second, while some service users may have higher education or relevant professional backgrounds, they tend to be average people in regard to conducting research and are unacquainted with the institutional context and research requirements. Here, for instance, the service users were the guests of the researchers and the research institution; therefore, it is part of the protocol of hospitality and collegiality to accommodate them during their visit so they feel welcomed. Hence, facilitating non-tokenistic user involvement entails providing enough knowledge to allow service users to contribute effectively regarding their personal and professional experiences, helping them feel valued in a usually unfamiliar work environment.

The described approaches can function as means in which to balance the existing asymmetry between researchers and collaborators, as well as to promote desired creativity and reflection during the participation process [11, 15]. The methods of pointers on large flip-over sheets with purposes and wishes for user involvement and matrices with priorities for goal achievement can serve as representational artefacts, in addition to documents, memos and minutes collected in folders or ring binders, which visualise the work and contribute to a common understanding. Such measures seem consistent with Morrison and Dearden’s concept of representational artefacts and ‘meaningful’, valuable participation, which has illuminated the significance of measures and arrangements. Several of the instruments implemented to facilitate cooperation have become common reference objects and artefacts, which exemplifies how representational artefacts can both embody the questions of professionals and be employed by user participants [11]. Regardless of form, the appropriate use of representational artefacts reflects the importance of mechanisms and mediums for discussing concrete ideas [11].

Furthermore, analysing and systematising the comments of the collaborators and undertaking ‘translation work’ is necessary for allowing the inputs to be used by the researchers. This will also be accessible to the collaborators, who can summarise their opinions and reflections. Such translation work can lead to the future participation process.

This article’s authors are aware that it may be appropriate to state that the collaborators participated on the researchers’ premises. This fact highlights that asymmetric relations between researchers and users must not be underestimated, especially when they concern the research quality. Even when individuals are motivated to participate, users can experience barriers to communicating their preferences, needs and values [8]. Many obstacles lie in the way of power during engagement processes, which affects how individuals participate and how their participation is legitimised as knowledge. Valuable engagement, therefore, begins by specifically addressing power dynamics [8]. Creating a balance of power does not mean ignoring researcher approaches and points of view. However, language play can place participants into relationships that limit what can be said and done. On the other hand, the creation of an alternative language games can serve as representational artefacts and provide other possibilities [11].

We believe that the collaborators cannot take advantage of participation opportunities if they lack the skills to interact in their settings. Morrison and Dearden argue that participants can sometimes worry about whether they count as participants in collaborative processes, doubting their skills to challenge the professionals on the board. Thus they need to be provided with education and training [11, 16].

The desire for non-tokenistic involvement calls for researcher responsibility to ensure that collaborators experience participation in a safe arena, and develop debate culture that can promote critical voices and allow participants to express their views. The fact that tensions and critical responses to researchers can still arise is significant. Underlying the establishment of a collaboration arena, differences still exist, but they are mediated by representational artefacts that allow participants to cooperate even if they represent different worlds. The language and the constructions expressed by researchers can complicate collaborator understanding or work as symbols of asymmetric relations. This case represents a certain cultural phenomenon. Nonetheless, this challenge also highlights the problems with simplifying language and expression and the difficulty in showing what research really is. The academic culture also requires preserving scientific precision and discussing such challenges together appears to be the best way to handle the issue.

The authors affirm that power inequalities between researchers and users continue. In fact, the tasks of acilitating and preparing for user engagement are both based on and maintain these disparities, with the inequalities the very basis of the need for user involvement. The collaborators in this research project wanted to hold their own meetings to shield themselves from possible researcher dominance, and they wanted the opportunity to discuss the research in other user forums. Non-tokenistic involvement means preparing opportunities for the users to discuss the research among themselves and their organisations.

Effective and valuable participation requires attention to specific methods of engagement so that they do not demand that participants express themselves in unfamiliar ways in order to be understood by researchers [11]. The user involvement in the analysed research project did not mean that the collaborators were co-researchers; rather, they were critical companions and discussants. However, they were involved in accordance with the principles of patient and public involvement as it appears in Gripp2-sf [26], and as evidenced throughout this article. They were not meant to be research experts but were expected to contribute with knowledge of a field of which they were most familiar: – the experiential knowledge of living with injuries.

## Limitations

This is a qualitative study of a single involvement process, which shows the views and the experiences of a particular group of researchers and collaborating service users, as well as their particular ways of demonstrating user involvement. Because ways of organising user involvement may differ [10], this study does not represent all the experiences of other stakeholders trying out non-tokenistic user involvement in research. Conducting auto-ethnography can also make it more difficult to perceive some aspects of the topic under investigation and be critical in the analyses even though it provides increased insight into other aspects. In this light, caution should be exercised in generalising the results.

## Conclusions

This analysis has shown strategies and measures that can facilitate non-tokenistic user involvement in research, suggesting that implementation means that organisational and practical aspects of the involvement are taken seriously and the design of the collaborative arena is emphasised. The use of representative artefacts that link the various participants in the collaborative group, according to this analysis, appear to have a significant impact on the implementation of non-tokenistic user involvement. Although this does not mean that the asymmetries in the interaction disappear, power imbalances become less prominent to allow valuable participation, relevant for the users. By distinguishing between defining the collaboration arena and designing for research counselling, the analysis has enabled elucidating the elements of the arrangements and identifying the empirical data that shows the importance of these elements. Overall, the study shows that the basis for non-tokenistic participation should be anchored in the researchers’ fundamental respect for participants and an honest engagement in their opportunities to contribute. This attitude promotes ways to avoid tokenistic user involvement and stimulate active engagement in research. The authors believe that this study can help researchers who want active participation and non-tokenistic involvement in research to find ways to facilitate and organise participation in their own studies and to hope that research aiming to contribute to this topic will increase in the coming years.

## Data Availability

Copies of the anonymised transcriptions of meetings are available on reasonable request, with the permission of the OsloMet - Oslo Metropolitan University, by contacting Tone Alm Andreassen.

## Abbreviation

Ph.D: Doctor of Philosophy, which is a postgraduate doctoral degree
